# Biofilm Formation of *Listeria monocytogenes* Strains Under Food Processing Environments and Pan-Genome-Wide Association Study

**DOI:** 10.3389/fmicb.2019.02698

**Published:** 2019-11-21

**Authors:** Bo-Hyung Lee, Sophie Cole, Stéphanie Badel-Berchoux, Laurent Guillier, Benjamin Felix, Nicolas Krezdorn, Michel Hébraud, Thierry Bernardi, Ibrahim Sultan, Pascal Piveteau

**Affiliations:** ^1^École Doctorale des Sciences de la Vie, Santé, Agronomie, Environnement, Université Clermont Auvergne, Clermont-Ferrand, France; ^2^BioFilm Control SAS, Biopôle Clermont Limagne, Saint-Beauzire, France; ^3^GenXPro GmbH, Frankfurt am Main, Germany; ^4^Maisons-Alfort Laboratory for Food Safety, Salmonella and Listeria Unit, University of Paris-Est, French Agency for Food, Environmental and Occupational Health & Safety (ANSES), Maisons-Alfort, France; ^5^UMR MEDiS, Institut National de la Recherche Agronomique (INRA), Université Clermont Auvergne, Clermont-Ferrand, France; ^6^MaIAGE, INRA, Université Paris-Saclay, Jouy-en-Josas, France; ^7^Agroécologie, AgroSup Dijon, INRA, Université Bourgogne Franche-Comté, Dijon, France

**Keywords:** biofilm, *Listeria monocytogenes*, pan-genome-wide association study, adhesion, intraspecies diversity, NaCl, nutrient deficiency, clonal complex

## Abstract

Concerns about food contamination by *Listeria monocytogenes* are on the rise with increasing consumption of ready-to-eat foods. Biofilm production of *L. monocytogenes* is presumed to be one of the ways that confer its increased resistance and persistence in the food chain. In this study, a collection of isolates from foods and food processing environments (FPEs) representing persistent, prevalent, and rarely detected genotypes was evaluated for biofilm forming capacities including adhesion and sessile biomass production under diverse environmental conditions. The quantity of sessile biomass varied according to growth conditions, lineage, serotype as well as genotype but association of clonal complex (CC) 26 genotype with biofilm production was evidenced under cold temperature. In general, relative biofilm productivity of each strain varied inconsistently across growth conditions. Under our experimental conditions, there were no clear associations between biofilm formation efficiency and persistent or prevalent genotypes. Distinct extrinsic factors affected specific steps of biofilm formation. Sudden nutrient deprivation enhanced cellular adhesion while a prolonged nutrient deficiency impeded biofilm maturation. Salt addition increased biofilm production, moreover, nutrient limitation supplemented by salt significantly stimulated biofilm formation. Pan-genome-wide association study (Pan-GWAS) assessed genetic composition with regard to biofilm phenotypes for the first time. The number of reported genes differed depending on the growth conditions and the number of common genes was low. However, a broad overview of the ontology contents revealed similar patterns regardless of the conditions. Functional analysis showed that functions related to transformation/competence and surface proteins including Internalins were highly enriched.

## Introduction

*Listeria monocytogenes* is a psychrotolerant Gram-positive, rod-shaped saprophytic bacterium. As a non-fastidious organism, it tolerates a range of stressful conditions. Its resistance to high osmolarity was demonstrated by growth up to 13% NaCl and survival under 40% NaCl (Liu et al., 2005; Shabala et al., 2008). *L. monocytogenes* can grow at low temperatures (Junttila et al., 2008) and its minimal growth temperature is expected to be at −2°C (Augustin et al., 2005). It withstands acidic and alkaline environments as well as low water activity conditions (Nolan et al., 1992; Koutsoumanis et al., 2003; Shen et al., 2016). Moreover, exposure to a stress factor can provide cross-adaptation to subsequent exposure to other stresses (Begley et al., 2002; Bergholz et al., 2012).

*Listeria monocytogenes* inhabits a broad range of habitats such as soil, silage, vegetation, sewage, and river (Welshimer and Donker-Voet, 1971; Garrec et al., 2003; Vivant et al., 2013; Linke et al., 2014). As an opportunistic foodborne pathogen, it is responsible for the life-threatening disease listeriosis. In the European Union (EU) in 2017, a total of 2,480 confirmed invasive human listeriosis were reported by 28 member states, corresponding to an EU notification rate of 0.48 cases per 100,000 population and a fatality rate of 13.8% (EFSA and ECDC, 2018). The presence of *L. monocytogenes* in the food processing environment (FPE) is a major burden for the food industry and a challenge for food safety. Indeed, evidence suggests that FPE is the most likely source of contamination of *L. monocytogenes* in different types of foods (Pérez-Rodríguez et al., 2008; McCollum et al., 2013) and growth of biofilms in FPE is considered to be one of the main sources of repeated bacterial food contaminations (Chmielewski and Frank, 2003; Giaouris et al., 2014; Colagiorgi et al., 2017). Biofilms are the predominant mode of microbial development in nature represented by microorganisms adhering to surfaces and growing as sessile communities. Stainless steel, polypropylene, glass or rubber are materials widely used in FPE that can support *L. monocytogenes* colonization (Mafu et al., 1990; Beresford et al., 2001; Chavant et al., 2002). When organized as biofilm, the self-produced extracellular polymeric matrix gives extra protection to bacteria from harsh environmental conditions such as desiccation, nutrient deprivation, or disinfectant treatment (Bridier et al., 2011; Esbelin et al., 2018). As a consequence, it is challenging to control bacterial contaminations in FPE. Despite an early belief that *L. monocytogenes* could only form monolayer biofilms, later studies demonstrated a varying degree of biofilm maturity (Borucki et al., 2003; Rieu et al., 2008; Guilbaud et al., 2015; Lee et al., 2017).

Pulsed-field gel electrophoresis (PFGE) has been the gold standard for the last few decades for epidemiology and intraspecific diversity analyses (Graves and Swaminathan, 2001; Félix et al., 2014). This typing method was the cornerstone for the evidence of persistent isolates. In parallel, multi-locus sequence typing (MLST) has emerged as a key method to investigate the genomic relatedness among *L. monocytogenes* isolates (Stessl et al., 2014). As a robust technic, MLST typing has the advantage of providing reproducible data across laboratories and it can be performed from whole genome sequence data (Cantinelli et al., 2013; Henri et al., 2016). High congruence between PFGE and MLST results allowed successful conversion of PFGE profiles into MLST data, thus enabling utilization of PFGE databases for investigation of population genetics, and clonal structure of *L. monocytogenes* (Maury et al., 2016; Félix et al., 2018).

In the past, attempts to correlate biofilm phenotype to serotype, origin or persistence gave conflicting results between studies and experimental conditions. Such comparisons can now be addressed at the level of the genome. Indeed, Genome-wide association study (GWAS) is a top-down approach that involves testing large numbers of genetic variants in a population of individual organisms with a given phenotype. The application of GWAS in microbiology has slowly emerged (Falush and Bowden, 2006; Falush, 2016) and application of GWAS to *Campylobacter* successfully identified genetic factors responsible for adaptation to different animal hosts (Sheppard et al., 2013). While most association studies are focusing on clinical phenotypes, GWAS dealing with food-related phenotypes was recently applied to *L. monocytogenes* traits associated to cold, salt, acid, and desiccation stresses (Hingston et al., 2017; Fritsch et al., 2019).

The aims of the present study were: (i) to investigate whether or not high biofilm forming capacity is related to persistence and/or prevalence of specific genotypes in FPE and related food products, (ii) to analyze intraspecific biofilm productivity in a set of environmental conditions, (iii) to compare biofilm phenotypes and genomes in order to identify modules of genes potentially linked to biofilm formation. Information on the prevalence of PFGE pulsotypes in food and food premises was extracted from a collection of 1667 isolates in order to select 58 isolates covering the genomic diversity and frequency of detection of *L. monocytogenes* in the food industry. Twenty prevalent as well as 19 rare isolates were selected, respectively, and 19 persistent isolates were included in the strain panel.

We evaluated the sessile growth at low temperature, under nutrition deprivation and exposure to salt, mimicking environmental conditions frequently encountered in food premises. To our knowledge, this is the first study that assessed adhesion capacity and biofilm formation at multiple levels including intergroups (persistent, prevalent, and rare isolates), lineages, serogroups, as well as genotypes. Pan-Genome-Wide Association Study (Pan-GWAS) further highlighted genes that could be associated with biofilm formation of *L. monocytogenes*.

## Materials and Methods

### *Listeria monocytogenes* Isolate Collection, Inoculum Preparation, and Growth Media

Fifty-eight isolates were selected from the strain collection of ANSES (French Agency for Food, Environmental and Occupational Health & Safety), in order to represent the genetic diversity of *L. monocytogenes* isolated from foods and FPE (Table 1). The selection criteria were first of all the phylogenetic position of each isolate determined by their pulsotype and MLST clustering (Henri et al., 2016). The second criterion was the frequency of isolation of the genotypes. These 58 isolates were grouped into lineage II (40 serogroup IIa and 3 serogroup IIc strains) and lineage I (5 serogroup IIb and 10 serogroup IVb strains). Three categories, prevalent, persistent, and rare isolates were considered according to the frequency of detection of the genotypes. The group of prevalent strains was composed of 20 isolates from the nine most dominant MLST clusters. Nineteen rare strains were selected from 15 minor clusters. Persistence was defined as isolates of the same genotype that were isolated at least three times over an extended observation period (1–5 years) from the same food premise. With these criteria, 19 isolates grouped within four MLST clonal complexes (CCs) each designated as subgroup A, B, C, and D were selected as persistent strains collected from France (subgroup A, B, and C) and Norway (subgroup D). As a whole, the strain collection was distributed into 27 different genotypes (CCs and sequence types, STs); 12 singletons, 9 genotypes represented by 2 isolates, 3 genotypes represented by 3 isolates, and 3 genotypes represented by 4, 7, and 8 isolates, respectively (Table 1).

**TABLE 1:** *Listeria monocytogenes* strains used in this study (Henri et al., 2016).

**Group**	**Subgroup^†^**	**Isolate**	**Type of source**	**Detail of source**	**Origin (date) of isolation**	**MLST**	**Serogroup^¥^**	**Lineage**
Persistent	A	1	Food	Smoked-salmon	France (16/11/2011)	CC121	IIa	II
		2	Food	Catering plate	France (29/6/2011)	CC121	IIa	II
		3	FPE	Wipping of ground	France (7/5/2014)	CC121	IIa	II
		4	FPE	Wipping of food contact surface	France (1/8/2015)	CC121	IIa	II
	B	5	FPE	Food conveyor	France (30/8/2011)	CC11	IIa	II
		6	FPE	Wipping of chain	France (24/9/2014)	CC11	IIa	II
		7	FPE	Chain bracket	France (1/12/2011)	CC11	IIa	II
	C	8	FPE	Sewer	France (1/6/2015)	CC155	IIa	II
		9	FPE	Machine before cleaning procedure	France (22/10/2014)	CC155	IIa	II
		10	FPE	Sewer on floor	France (1/5/2015)	CC155	IIa	II
		11	Food	Sandwich (ham and butter)	France (1/8/2015)	CC155	IIa	II
	D	12	FPE	Factory environment	Norway (21/12/2006)	CC7	IIa	II
		13	FPE	Factory environment	Norway (7/11/2007)	CC7	IIa	II
		14	FPE	Slicer machine before use	Norway (21/11/2007)	CC7	IIa	II
		15	FPE	Factory environment	Norway (25/10/2009)	CC7	IIa	II
		16	FPE	Factory environment	Norway (7/11/2009)	CC7	IIa	II
		17	FPE	Factory environment	Norway (21/11/2009)	CC7	IIa	II
		18	FPE	Factory environment	Norway (15/3/2010)	CC7	IIa	II
		19	FPE	Factory environment	Norway (22/9/2011)	CC7	IIa	II
Prevalent		1	Food	Sandwich (ham and cheese)	Essonne, France (15/5/2008)	CC121	IIa	II
		2	Food	Sliced tomato	Essonne, France (15/5/2008)	CC121	IIa	II
		3	Food	Ham	Val-de-Marne, France (7/3/2011)	CC121	IIa	II
		4	Food	Cheese (Gouda)	France (6/6/2003)	CC9	IIc	II
		5	Food	Spinach	Cote-d’Or, France (2/5/2005)	CC9	IIc	II
		6	Food	Sausage (Merguez)	France (25/8/2005)	CC9	IIc	II
		7	Food	Frozen onions	Vendee, France (20/1/2009)	CC8	IIa	II
		8	Food	Sausage meat	Guadeloupe, France (25/5/2010)	CC8	IIa	II
		9	Food	Smoked-haddock	Paris, France (25/10/2002)	CC2	IVb	I
		10	Food	White chocolate mousse	Alpes-Maritimes, France (1/3/2007)	CC2	IVb	I
		11	Food	Grilled vegetables	Essonne, France (26/9/2005)	CC1	IVb	I
		12	Food	Goat milk	Indre, France (16/12/2005)	CC1	IVb	I
		13	Food	Vacuum-packed goose breast fillet	Landes, France (1/7/2003)	CC3	IIb	I
		14	Food	Pastrami	Martinique, France (17/3/2010)	CC3	IIb	I
		15	FPE	Rag for surface wipping	Dordogne, France (28/1/2008)	CC4	IVb	I
		16	Food	Red pepper	Ille-et-Vilaine, France (19/5/2006)	CC4	IVb	I
		17	Food	Salad (Piedmontese)	Guadeloupe, France (25/5/2010)	CC5	IIb	I
		18	Food	Sausage meat with vegetable	Savoie, France (20/8/2008)	CC5	IIb	I
		19	Food	Potatoes	Gironde, France (16/3/2006)	CC6	IVb	I
		20	Food	Frozen tomatoes	Vendee, France (20/1/2009)	CC6	IVb	I
Rare		1	Food	Cheese (Munster)	Yvelines, France (5/1/2012)	CC451	IIa	II
		2	Not Known	Not Known	Ille-et-Vilaine, France (6/5/2009)	CC14	IIa	II
		3	Food	Cheese	Not Known	CC177	IIa	II
		4	Food	Salmon steak	Eure, France (17/7/2003)	CC19	IIa	II
		5	Food	Cheese	Not Known	CC199	IIa	II
		6	Food	Cheese	Herault, France (9/12/2008)	CC20	IIa	II
		7	Food	Cheese (Cantal)	Cantal, France (7/5/2009)	CC21	IIa	II
		8	Food	Salmon	Finistere, France (26/9/2005)	CC220	IVb	I
		9	Food	Cheese	Not Known	CC26	IIa	II
		10	Food	Cheese	Orne, France (29/4/2009)	CC26	IIa	II
		11	Food	Cheese	Orne, France (7/7/2009)	CC26	IIa	II
		12	Food	Salad (rice, corn, pepper, and ham)	Loiret, France (5/5/2011)	CC31	IIa	II
		13	FPE	Not known	Not Known	CC31	IIa	II
		14	Food	Sandwich (smoked-salmon and mimolette cheese)	Essonne, France (1/3/2006)	CC315	IVb	I
		15	Food	Cheese	Corse, France (9/10/2008)	CC412	IIa	II
		16	Food	Soy bean sprouts	Vendee, France (21/4/2011)	ST200	IIa	II
		17	Food	Cheese (Morbier)	Jura, France (5/9/2012)	ST517	IIb	I
		18	Food	Not Known	Not Known	ST13	IIa	II
		19	Food	Duck liver (foie gras)	Landes, France (8/3/2007)	ST13	IIa	II

*^†^Isolates belonging to each subgroup were isolated from the same food premise and presumed to be persistent clones. ^¥^Serogroup IIa (serotype 1/2a and 3a), IIb (1/2b, 3b and 7), IIc (1/2c and 3c), and IVb (4b, 4ab, 4d, and 4e) by a multiplex PCR assay (Doumith et al., 2004).*

A working stock was prepared in brain-heart infusion (BHI) broth (Laboratorios Conda, Spain) with 8.3% glycerol (Sigma-Aldrich, France) and stored at −20°C. Bacteria were sub-cultured twice on BHI agar at 37°C. Overnight grown colonies were harvested and homogenized in BHI or 1:10 diluted BHI (dBHI) media. When required, 0.85% w/v NaCl (Sigma-Aldrich) was added to the growth medium.

### Biofilm Quantification With Microtiter Plate Assay (MPA)

The microtiter plate assay was performed as previously described by Lee et al. (2017) with slight modifications. Briefly, overnight grown colonies were diluted to obtain an OD_600_ of 0.1 in each growth medium and 200 μl of bacterial solution was transferred in triplicate wells in 96-well microplates. Sterile media were added as a negative control. The effect of three factors on biofilm formation was tested: nutrient availability (BHI or dBHI), salt content (0 or 0.85% w/v NaCl), and temperature (10 or 37°C). Microplates were incubated statically for 24 h at 37°C or 10°C. Plates were inverted and the media and planktonic cells were removed by gentle tapping. To remove loosely attached bacteria, wells were washed twice with 300 μl of sterile saline solution (8.5 g NaCl per liter). Then biofilms were fixed with 300 μl of 96% v/v ethanol (Sigma-Aldrich) for 20 min and air-dried completely at room temperature after removal of the ethanol. For staining bacterial biomass, 220 μl of 0.1% w/v crystal violet (CV; Merck, Germany) solution was added per well and plates were incubated statically for 30 min. Then the solution was removed by sharply tapping plates upside down. Wells were washed 3 times with 300 μl of saline and air-dried completely before filling with 150 μl of 33% v/v acetic acid (Sigma-Aldrich). Plates were placed on a plate shaker with slight agitation for 10 min to completely dissolve CV and get homogenized solutions. The amount of destained CV was determined by reading OD_600_ in a microplate reader (EL800, BioTek, United States).

At least three experiments were performed for each condition with independently grown bacterial cultures.

### Assessment of Adhesion Capacity: Biofilm Ring Test^®^ (BRT)

The biofilm ring test assay (KitC004, BioFilm Control, France) was carried out in polystyrene 96-well microplates as described by Chavant et al. (2007) with slight modifications.

Initial inoculums to an OD_600_ of 0.2 were prepared in BHI and dBHI broths, each containing magnetic beads (Toner4) at a final concentration of 10 μl ml^–1^. A 10-fold dilution, from OD_600_ 0.2 to 0.0002, was performed to increase the detectable range of phenotypic changes. In a microplate, 200 μl of suspension was transferred into triplicate wells along with negative controls composed of BHI or dBHI broths with magnetic beads. Plates were incubated statically at 10 or 37°C. After 5 h incubation, few drops of Liquid Contrast (inert and non-toxic white oil) were deposited in wells and plates were placed on the Block Test for 1 min to apply magnetic fields at the center of each well. The bottom of plates was scanned with the Plate Reader and analyzed by BFC elements 3^®^ software to obtain the numeric values of each well called the BioFilm Index (BFI) ranging from 0 to 20. With this measurement, absence of biofilm in a well results in high mobility of magnetic beads and a BFI around 20. In contrast, immobilization of beads by sessile cells results in a lower BFI or zero value.

At least three experiments were performed for each condition with independently grown bacterial cultures.

### Scanning Electron Microscopy (SEM) of Biofilms

Overnight grown cells were suspended to obtain an OD_600_ of 0.1 in 4 different growth media: BHI or dBHI broths supplemented with 0 or 0.85% NaCl. Seven milliliters of each bacterial suspension were poured into a Petri dish (55-mm diameter) containing a flat stainless-steel coupon (AISI 304, mean roughness = 0.064, 2.5 cm × 1 cm). After 24 h incubation at 37°C under static conditions, coupons were gently washed twice with sterile saline solution. Sessile cells and biofilms were fixed with a solution of 3% glutaraldehyde in 0.2 M cacodylate buffer (pH 7.4) then coupons were kept at 4°C for a minimum of 1 h to overnight. Before dehydration steps, coupons were washed three times with cacodylate buffer for 15 min each. Coupons were dehydrated using a graded ethanol series (70, 90, and 100%) three times, 15 min each, and dehydrated further in a 50:50 mixture of ethanol:hexamethyldisilazane (HMDS, Delta microscopies, France) three times for 10 min each. Samples were immersed twice for 10 min each in pure HMDS followed by air-drying at room temperature and sputter-coated with gold (JFC-1300, JEOL, Japan) and observed with a scanning electron microscope (JEOL 6060LV, JEOL).

### Pan-Genome-Wide Association Study (Pan-GWAS)

This analysis was performed on the 57 strains for which the whole genome sequence data were available from a previous study (Henri et al., 2016). The paired-end reads of the strains used in the study are available under the ENA bio-projects PRJEB15592 and PRJEB32254^1^^,^^2^.

Total biomass measured by MPA under eight different growth conditions were classified into binary phenotypes, strong or weak biofilm formers. Strains were clustered based on their MPA OD_600_ values using a function hclust (RStudio version 3.4.2) with the complete linkage method for hierarchical clustering.

Pan-GWAS was performed based on the accessory gene content of the 57 *L. monocytogenes de novo* assemblies. First, draft genomes were obtained using the SPAdes algorithm (Bankevich et al., 2012) after quality check of Illumina reads using FastQC (Andrews, 2010). Then, whole genome annotation was carried out using Prokka (Seemann, 2014) with default parameters. Using the assemblies as an input Prokka produced GFF3-files, including sequences and annotations, which were used to extract the pangenome of the 57 *L. monocytogenes* isolates with Roary (Page et al., 2015). Finally, gene-based GWAS was performed using Scoary (Brynildsrud et al., 2016) by following the instructions provided on https://github.com/AdmiralenOla/Scoary. Scoary assessed presence/absence patterns of genes in binary phenotypes and the genes with significant association (*p* < 0.05) were reported. As proposed by the Scoary pipeline, population structure was accounted for in order to control the confounding population stratification. Pairwise comparisons were performed based on the phylogenetic tree (see section “Phylogenetic Analysis”) for population structure correction. Percentage of strains between strong versus weak biofilm formers carrying each gene in their genome was calculated. For example, % of a gene in strong biofilm formers was determined as follows: (number of strains carrying the gene in their genomes among strong biofilm formers) × 100/(total number of strains grouped into strong biofilm formers). Information regarding gene name, protein name, accession number and gene ontology (GO) were retrieved from the Universal Protein Resource database^3^. A limited percentage of sequences (0.79%) with no BLAST hit was excluded from further analysis. To obtain a visualized dataset of the selected genes, GO enrichment analysis (GO-slim) was performed using PANTHER Version 14.0^4^. Selected sequences were annotated according to *L. monocytogenes* EGDe genome (Refseq accession NC_003210.1) and assessed for functional classification retrieved from ListiList^5^.

### Phylogenetic Analysis

A variant calling analysis was performed using iVARCall2 (Felten et al., 2017) by which paired-end reads were aligned against a reference genome to identify single-nucleotide polymorphisms (SNPs) and InDels by local *de novo* assembly. The complete sequence of *L. monocytogenes* EGDe was used as the reference genome. Phylogenomic reconstruction based on core-genome SNPs was carried out with randomized axelerated maximum likelihood (Stamatakis, 2014). The phylogenetic inference was performed with bootstrap analysis and searching for the best-scoring Maximum Likelihood tree with general time-reversible model of substitution and the secondary structure 16-state model.

### Statistical Analyses

One-way analysis of variance (ANOVA) with Tukey’s multiple comparison test was applied to determine statistically significant differences in the level of adhesion and biofilm production within lineages, serogroups, groups (persistent, prevalent, and rare) and genotypes. Dunnett’s multiple comparison test was applied for testing the significance of changes in biofilm production caused by alteration of growth conditions (T°C or % NaCl) in comparison to the standard BHI or dBHI condition. Multiple *t*-test was applied to assess the statistical significance of changes in adhesion measured by BRT. All differences are reported at a level of significance of 0.01 or 0.05.

## Results

### Environmental Conditions Affect Biofilm Production

**FIGURE 1:**
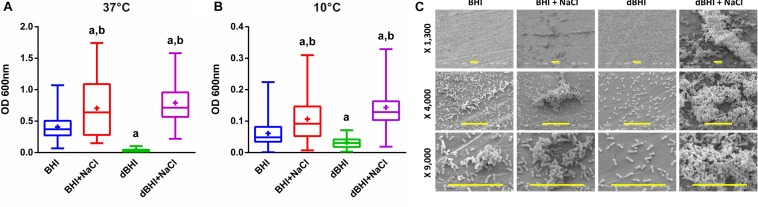
Effect of various growth conditions on biofilm formation. Total biomass of all 58 isolates quantified by MPA at **(A)** 37°C and **(B)** 10°C are plotted. Whiskers extend to minimum and maximum values, and the horizontal line and the plus symbol (+) in the box represent the median and mean values, respectively. Statistical significance was assessed using One-way ANOVA and Dunnett’s multiple comparison tests, a, *p* < 0.01 compared to BHI; b, *p* < 0.01 compared to dBHI. **(C)** Examples of SEM observation at low, middle, and high magnifications (row) for strain 13 (persistent group) biofilm formation at 37°C according to the growth conditions. Scale bars: 10 μm. MPA result of this strain can be found in Supplementary Figure S1.

Biofilm production by 58 isolates was evaluated at 37°C, the optimal growth temperature for *L. monocytogenes* and at 10°C, a low temperature mimicking conditions in the FPE. At each temperature, four culture media were tested: BHI as a control, dBHI representing a nutrient-deprivation, and both media with addition of 0.85% NaCl to assess impact of salt. Temperature played a major role in determining the total sessile biomass; in general, biofilm production was more than five times greater at 37°C than at 10°C with the exception of dBHI broth in which the total biomass produced at both temperatures was similar (Figures 1A,B). Similar trends upon changes in media composition were observed at both temperatures. Compared to full-strength BHI, nutrient deprivation (dBHI) induced a significant decrease in total biomass and notably the effect was more prominent at 37°C. In contrast, addition of 0.85% NaCl in BHI or dBHI induced a significant increase in biofilm production. This effect was significantly stronger in dBHI than in BHI. Supplementation of dBHI with NaCl resulted in 23.4 and 4.5 times higher total biomass at 37°C and 10°C, respectively, while 1.7 times increase was observed when NaCl was added to BHI at both temperatures. It was noticeable that the combination of two factors, addition of salt and deprivation of nutrient, exerted a non-linear positive effect on biofilm production. Based on data from the literature (Takahashi et al., 1981) addition of 0.85% NaCl to dBHI increased osmolarity from 36 to 327 mOsm/L, an estimate comparable to the osmolarity of BHI (360 mOsm/L). However, the bacteria produced significantly more biomass in dBHI + NaCl compared to that in BHI suggesting that nutrient deprivation stimulates biofilm production provided that appropriate osmotic environment is supplied.

Scanning electron microscopy observations of strain 13 (persistent group) showed several biofilm structures from monolayers of cells to mature 3-D biofilms depending on the environmental conditions (Figure 1C). Cells grown in BHI uniformly colonized the surface with small cellular aggregates and addition of salt increased the volume and frequency of cell clusters. When deprived of nutrients (dBHI), the surface was only sparsely colonized with no visible aggregate. However, addition of salt in dBHI dramatically improved biofilm construction and dense, three-dimensional mature biofilms were observed.

### Biofilm Formation of Persistent, Prevalent, and Rare Isolates

**FIGURE 2:**
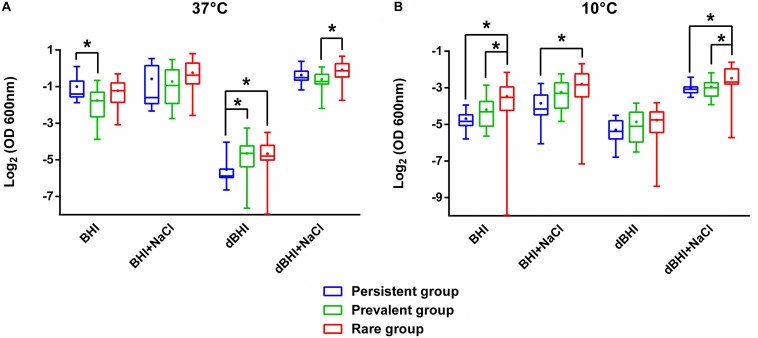
Comparative analysis of biofilm formation among persistent, prevalent, and rare isolates. Total biomass quantified by MPA at **(A)** 37°C and **(B)** 10°C are plotted. Whiskers extend to minimum and maximum values, and the horizontal line and the dot in the box represent the median and mean values, respectively. Data were analyzed using One-way ANOVA and Tukey’s multiple comparisons test. ^∗^*p* < 0.05.

To test the hypothesis whether higher biofilm forming capacity could underlie persistence or frequent isolation of specific genotypes, we compared the biomass produced under diverse conditions among persistent (*n* = 19), prevalent (*n* = 20), and rare (*n* = 19) isolates (Figure 2). Interestingly, isolates of the rare group formed significantly more biofilms than isolates of the other two groups under numerous conditions. On the contrary, persistent isolates showed significantly higher biofilm formation only in BHI at 37°C where their biofilm production was higher than that of prevalent isolates. Similarly, prevalent isolates did have significantly higher biofilm production only when compared to persistent isolates in dBHI at 37°C. These observations suggest that, under the conditions tested, biofilm production cannot discriminate persistence or distribution frequency of genotypes in foods and FPE.

### Association of Biofilm Production With Phylogenetic Division

**FIGURE 3:**
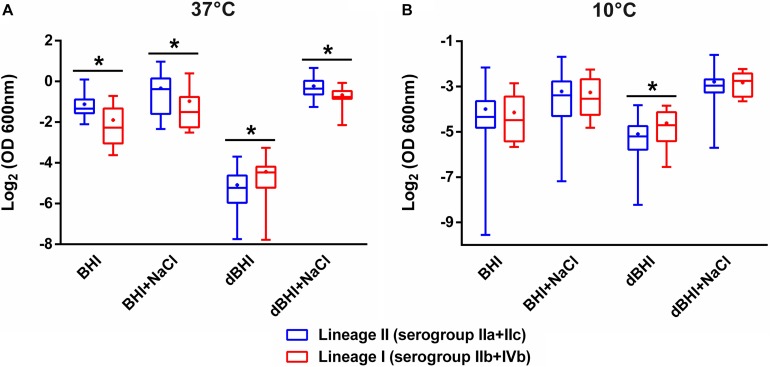
Comparative analysis of biofilm formation between lineages II and I. Total biomass quantified by MPA at **(A)** 37°C and **(B)** 10°C are plotted. Whiskers extend to minimum and maximum values, and the horizontal line and the dot in the box represent the median and mean values, respectively. Data were analyzed using One-way ANOVA and Turkey’s multiple comparison tests. ^∗^*p* < 0.05.

Associations between phylogenetic attributes and biofilm formation were evaluated (Figure 3). Lineage II strains were statistically more efficient than lineage I strains in producing biofilms under three (BHI, 37°C; BHI + NaCl, 37°C; dBHI + NaCl, 37°C) out of eight conditions. However, opposite results were observed after incubation in dBHI whatever the temperature. No significant differences were observed under the other conditions tested. Comparative analysis at the level of serogroups were concordant with the differences observed with lineages (Supplementary Figure S2). At 37°C, serogroup IVb strains produced significantly less biofilm than serogroup IIa and/or IIb strains under three conditions (BHI, BHI + NaCl, and dBHI + NaCl). On the contrary, IVb strains formed more biofilm in dBHI at 37°C. However, some significant differences were also detected within lineage I, concurrently between serogroups IIb and IVb strains.

### Intra- and Inter-Genotype Variations in Biofilm Phenotype

**FIGURE 4:**
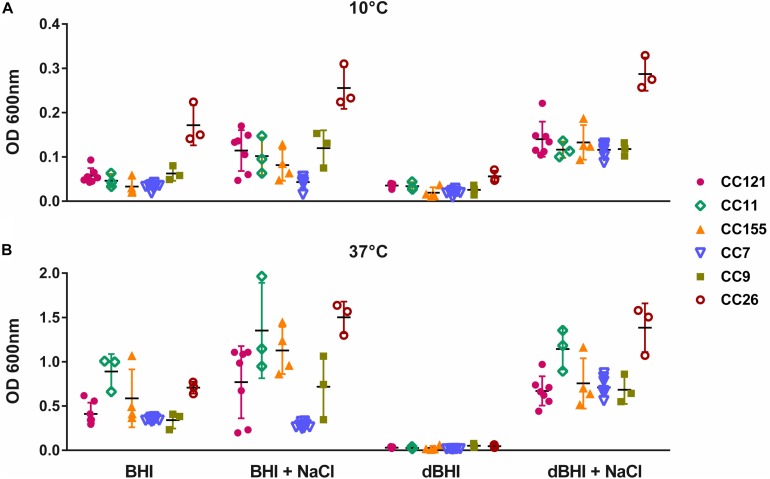
Inter- and intra-genotype variation in biofilm formation. MPA results are shown at **(A)** 10°C and **(B)** 37°C. Isolates belonging to six genotypes, each represented by at least three isolates are plotted; CC121 (*n* = 7), CC11 (*n* = 3), CC155 (*n* = 4), CC7 (*n* = 8), CC9 (*n* = 3), and CC26 (*n* = 3). Each symbol represents one isolate; vertical lines indicate SD and horizontal bars the mean values. Data were analyzed using One-way ANOVA and Turkey’s multiple comparison tests, *p* < 0.05. CC26 formed statistically more biofilms than other genotypes in all conditions at 10°C and in dBHI + NaCl at 37°C.

The genotypes represented by at least 3 isolates (28 isolates in total) were selected to assess intra- and inter-genotypic variances. Significant intra-genotype variations were observed except for CC7 and CC26. At least one pair of isolates within genotypes CC121, CC11, CC155, and CC9 revealed statistically different results under one or more growth conditions (Supplementary Figure S3). Considering inter-genotype variations, total biomass produced by isolates belonging to CC26 were distinctively higher than other 5 CCs under all four different conditions at 10°C (Figure 4A). At 37°C differences were significant under only one condition (dBHI + NaCl) implying that the cold temperature enhanced the biofilm forming trait of genotype CC26 (Figure 4B).

### Nutrient Deficiency Induces Higher Adhesion

**FIGURE 5:**
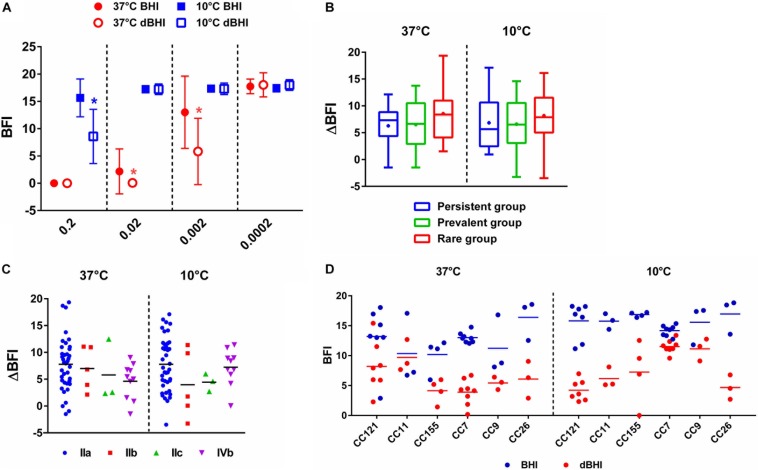
Enhanced adhesion *of L. monocytogenes* upon nutrient stress measured by BRT. **(A)** BRT results of 58 isolates under four different conditions (symbols) at different inoculum concentrations (*X*-axis). Data are presented as mean ± SD and statistical significance was determined by multiple *t*-test. ^∗^*p* < 0.05 compared to BHI at each temperature. Delta BFI (ΔBFI) of **(B)** three groups, persistent, prevalent, and rare and **(C)** serogroups. ΔBFI was calculated by subtracting BFI of cells incubated in dBHI from that in BHI. Whiskers extend to minimum and maximum values, and the horizontal line and the dot in the box represent the median and mean values, respectively. **(D)** BFI values from BHI and dBHI are shown for six genotypes, each containing more than three isolates. Each dot represents one isolate and horizontal bars the mean value. **(B–D)** BRT results of cells inoculated at OD_600_ of 0.002 were used for 37°C and 0.2 for 10°C.

Adhesion is the first step of biofilm formation corresponding to attachment of bacterial cells to biotic or abiotic surfaces. It was measured by the BFI, a parameter inversely proportional to the level of adhesion. BFI values of nutrient deprived cells (dBHI) were lower than those of control cells (BHI) demonstrating enhanced adhesion upon exposure to nutrient starvation for 5 h (Figure 5A). The positive effect of nutrient starvation on adhesion was observed regardless of the incubation temperature. At 37°C, the two highest inoculums gave results out of range (BFI = 0). Full coverage of the bottom of the plate by sessile cells resulted in nearly complete blockage of the beads. However, significant differences between BHI and dBHI were observed in a lower inoculum (OD_600_ of 0.002; app. 4E^+06^ cells/ml). By contrast, at 10°C, only the highest inoculum (OD_600_ of 0.2; app. 4E^+08^ cells/ml) showed the impact of nutrient stress while no adhesion could be recorded with lower inoculums due to insufficient number of cells.

To further compare the extent of change in adhesion, ΔBFI was calculated by subtracting BFI of cells incubated in dBHI from that in BHI. No significant differences were found among the three groups (persistent, prevalent, and rare) and serogroups (Figures 5B,C) suggesting that enhanced adhesion under nutrient deprivation was a universal phenotype transition. Furthermore, no evidence of genotype-specific predisposition to adhesion was recovered (Figure 5D). In summary, enhanced adhesion of *L. monocytogenes* was a global cellular response to hypo-osmotic shock triggered by nutrient deprivation.

### Phylogenetic Analysis of Relative Biofilm Productivity

**FIGURE 6:**
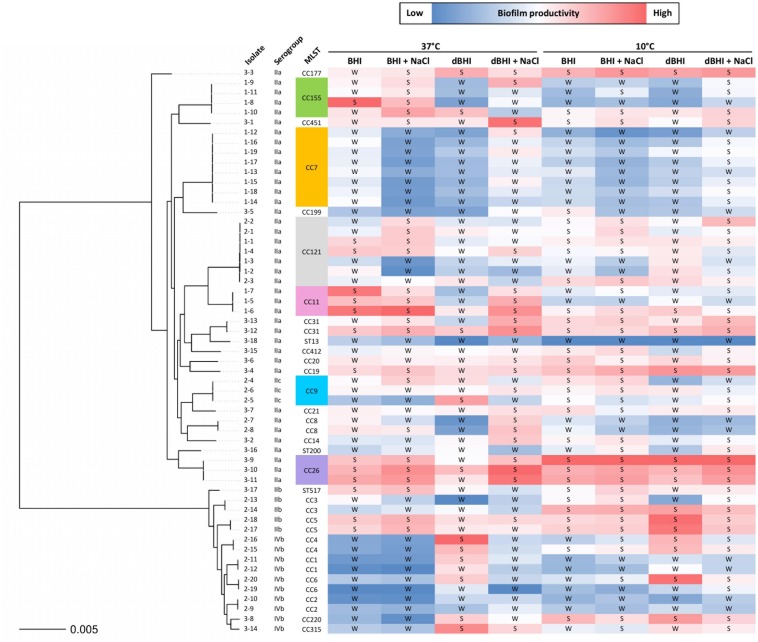
Phylogenetic tree of 57 *L. monocytogenes* isolates with relative biofilm productivity. A color scale is applied to MPA results within each growth condition to reflect biofilm productivity. The isolate names are shown as group number (1, persistent; 2, prevalent; 3, rare) hyphened with isolate numbers. Binary transformation of the isolates based on MPA results is indicated as S, strong and W, weak biofilm former.

The overall results of biomass produced by 57 isolates across all experimental conditions were transformed into binary phenotypes of strong or weak biofilm formers and grouped according to their phylogenetic positions (Figure 6). The results showed that relative biofilm productivity varied inconsistently within each isolate depending on the growth conditions except for some isolates. For example, isolates belonging to CC7 and CC26 demonstrated, respectively, lower, and higher biofilm productivity across all the conditions. This was consistent with homogenous intra-genotype traits observed with theses CCs under each condition (Supplementary Figure S3). On the other hand, high variation in biofilm productivity was observed between some phylogenetically closely related isolates, including those of the same genotypes.

By binary transformation, only 2 and 6 isolates were consistently classified as strong and weak biofilm formers, respectively, regardless of the growth conditions.

### Putative Determinants of Biofilm Formation Detected by Pan-GWAS

**FIGURE 7:**
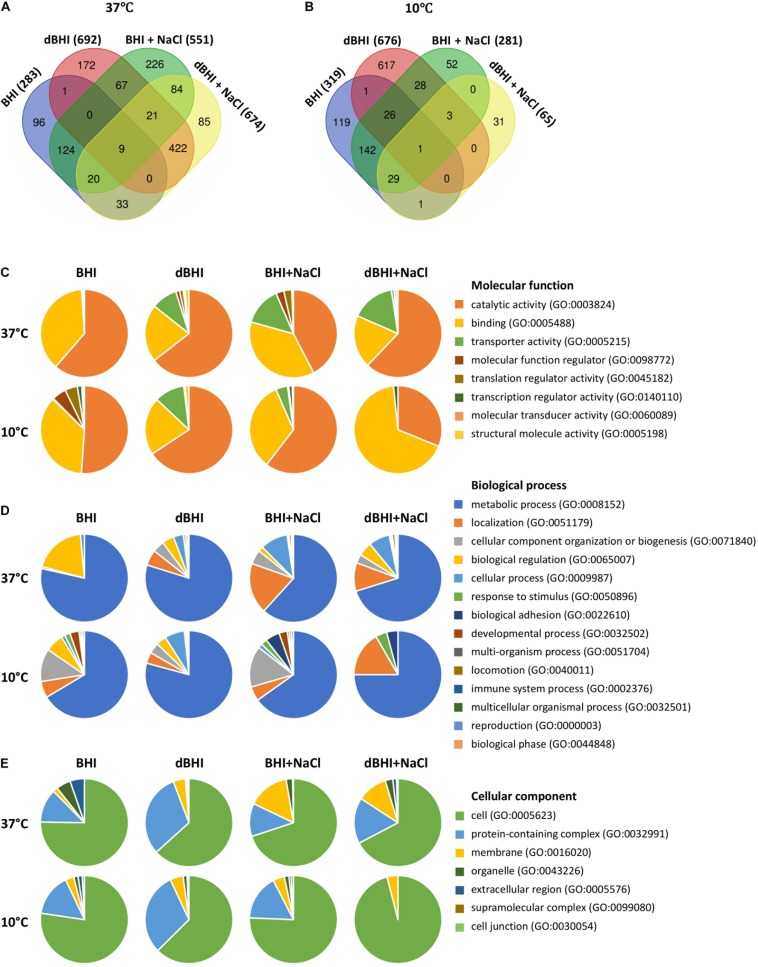
Genes identified by pan-genome-wide association study (pan-GWAS) and gene ontology (GO) analysis under different growth conditions. Venn diagrams show number of genes related to each condition at **(A)** 37°C and **(B)** 10°C. PANTHER derived GO-slim categories for **(C)** molecular function, **(D)** biological process, and **(E)** cellular component are presented in pie charts.

From the available pan-genome data of 57 strains, pan-GWAS identified genes whose presence or absence was significantly related to the binary biofilm phenotype (*p* < 0.05). These sets of genes varied greatly according to the growth conditions. Because temperature is a major environmental signal triggering regulation cascades in *L. monocytogenes*, the sets of genes identified at 37°C and 10°C were compared. The total number of genes identified from experiments performed in BHI, dBHI, BHI + NaCl, and dBHI + NaCl was 283, 692, 551, and 674, respectively, at 37°C, and 319, 676, 281, and 65, respectively, at 10°C (Figures 7A,B and Supplementary Tables S1–S8). Among the 1360 genes reported at 37°C, only 50 genes (3.68%) were found in more than three conditions. Likewise, among 1050 genes identified at 10°C, 59 genes (5.62%) were found in more than three conditions. Globally, considering all eight conditions, 37% of the genes identified by pan-GWAS had no predicted protein function. In order to compare the occurrence of pan-GWAS-identified genes between weak and strong biofilm formers, the percentage of strains carrying the genes was calculated (Supplementary Tables S1–S8). Depending on the genes and conditions, the difference in occurrence between weak and strong biofilm formers varied from 15 to 63% and the overall average difference on the full set of pan-GWAS genes was 29%.

PANTHER GO-Slim analysis was applied to investigate the distribution of high-level GO terms under each condition. Total 34, 273, 122, and 286 genes at 37°C, and 80, 262, 46, and 10 genes at 10°C were enriched in BHI, dBHI, BHI + NaCl, and dBHI + NaCl, respectively. Among the GO category “Molecular function,” the most enriched classes were “catalytic activity” (GO:0003824) followed by “binding” (GO:0005488), and “transporter activity” (GO:0005215) (Figure 7C). In the category “Biological process,” the “metabolic process” (GO:0008152) was overrepresented followed by “localization” (GO:0051179), “cellular component organization or biogenesis” (GO:0071840), and “biological regulation” (GO:0065007) classes (Figure 7D). In the “Cellular component” category, the most prevalent class was “cell” (GO:0005623) followed by “protein-containing complex” (GO:0032991) and “membrane” (GO:0016020) classes (Figure 7E).

**FIGURE 8:**
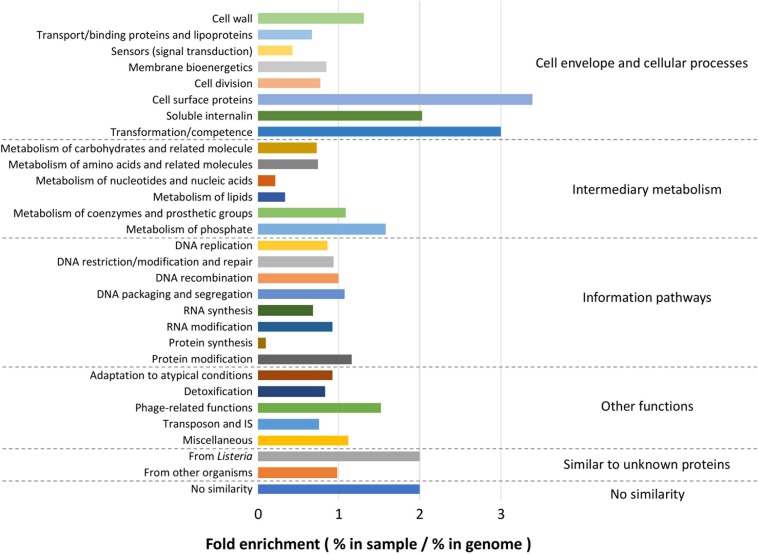
Functional enrichment analysis. Among the total list of genes identified by pan-GWAS, 2105 genes were annotated to *L. monocytogenes* EGDe and compared to the functional classification retrieved from ListiList (http://genolist.pasteur.fr/ListiList/). Fold enrichment of each functional category was performed as follow: Fold enrichment = % in annotated genes/% in the whole genome of EGDe. Functional categories are presented in super- and subclass on the right and left of the figure, respectively.

Because the enriched GO terms were similar regardless of the growth conditions, the whole list of genes identified by pan-GWAS was subjected to functional analysis. In total, 2105 genes (genes appeared in several conditions were counted each time for enrichment) were annotated to the genome of *L. monocytogenes* EGDe. Within each functional category, fold enrichment was calculated as (% in identified genes)/(% in whole genome of EGDe) (Figure 8). The most highly enriched functional categories belonged predominantly to “Cell envelope and cellular processes,” for example, “cell surface proteins” (3.39-fold enriched), “soluble internalin” (2.03-fold enriched), “transformation/competence” (3.00-fold enriched) and “cell wall” (1.31-fold enriched). In “Intermediary metabolism” category, “metabolism of phosphate” (1.58-fold enriched) was overrepresented while “metabolism of nucleotides and nucleic acids” (0.22-fold enriched) and “metabolism of lipids” (0.33-fold enriched) were comparatively underrepresented. Among the other functional categories, “phage-related functions” (1.52-fold enriched), “similar to unknown proteins from listeria” (2.00-fold enriched) and “no similarity” (1.9-fold enriched) were overrepresented. On the other hand, “protein synthesis” (0.09-fold enriched) was underrepresented.

## Discussion

Complex mechanisms regulate bacterial sessile growth and biofilm formation represented by adhesion, maturation and dispersal steps, each affected by intrinsic and extrinsic factors. In this study, 58 *L. monocytogenes* isolates from foods and FPE were evaluated for adhesion and sessile biomass production. Under all conditions studied, all isolates adhered and developed as biofilms. Diverse growth conditions reflecting environmental conditions of foods and FPE such as changes in salt content, nutrient availability and temperature significantly affected biofilm production. Temperature was the most influencing factor for biofilm production as observed in previous studies (Di Bonaventura et al., 2008; Rodrigues et al., 2009; Kadam et al., 2013). Experiments were performed at 10°C to mimic food processing conditions and at 37°C, although of lesser relevance to FPE, as a standard laboratory growth temperature. The lower growth rate of *L. monocytogenes* at temperature below optimal range can explain the significant differences between the biomass produced at optimal (37°C) and cold (10°C) temperatures during the 24 h of incubation. It needs to be investigated whether or not similar mature biofilms would be produced at 10°C if the incubation period was prolonged as shown for other strains (Chavant et al., 2002; Zameer et al., 2010).

In food industry, salt is the most widely applied natural preservative for foods especially in processed foods including RTE products (Institute of Medicine (US) Committee on Strategies to Reduce Sodium Intake, 2010). *L. monocytogenes* efficiently adapts to changes in water activity in the surrounding environment (Sleator et al., 2003) and NaCl was reported to induce biofilm production. Higher biomass was obtained when tryptic soy broth (TSB) containing 0.6% yeast extract was supplemented with increasing quantities of NaCl from 0.5% to 2% at 22.5, 30, and 37°C (Pan et al., 2010). Similarly, addition of 2–5% NaCl in TSB caused a dramatic increase in aggregation of *L. monocytogenes* (Jensen et al., 2007). The present study demonstrated that addition of salt at saline concentration (0.85% NaCl) induced a significant increase in biofilm formation under both nutrient rich (BHI) and poor (dBHI) conditions. Besides, our results disclosed for the first time a dramatic non-linear cumulative effect of salt addition and nutrient deprivation on biofilm production. Indeed, the positive effect of NaCl on biofilm maturation was significantly intensified by nutrient deprivation.

*Listeria monocytogenes* is likely to encounter nutrient deprivation or hypo-osmotic stress in FPE. Bacteria have evolved several mechanisms to avoid cell lysis caused by the massive influx of water into the cytoplasm under sudden hypo-osmotic shock. These include mechanosensitive or stretch-activated channels that mediate efflux of cytoplasmic solutes and water channels such as aquaporins (Sleator and Hill, 2002). Our study found that when cells were exposed to sudden nutrient deprivation, the initial adhesion step was enhanced at both optimal (37°C) and cold (10°C) temperatures. This finding is in agreement with our previous study that demonstrated enhanced adhesion of *L. monocytogenes* upon sudden cold stress (Lee et al., 2017). Both studies were aligned in the fact that the increased adhesion upon sudden stresses was not related to the serotype, origin or genotype of the strains as previously reported in the literature (Kalmokoff et al., 2001). Physicochemical properties such as hydrophobic interactions and interfacial forces between bacteria and substrata influence cell attachment (Renner and Weibel, 2011). Our previous study found correlations between cell surface properties and adhesion levels (Lee et al., 2017). Similarly, the sudden hypo-osmotic shock accompanied by nutrient stress could have triggered alterations in cell surface properties as a response to the stress, resulting in the increased adhesion. However, the mechanisms behind this observation remain to be elucidated. Furthermore, the increased adhesion upon nutrient deficiency did not lead to mature biofilm suggesting that each step of biofilm formation is differently affected by environmental factors.

*Listeria monocytogenes* shows a highly heterogeneous population. To date, studies that evaluated intraspecific diversity of biofilm phenotype focused on associating phenotypes with lineages, serotypes or origins of the strains. However, conflicting results were reported among studies (Djordjevic et al., 2002; Borucki et al., 2003; Nilsson et al., 2011; Doijad et al., 2015) and the inconsistencies could be ascribed to differences in experimental setups as well as selected strains (Kadam et al., 2013). Similarly, in the present study, significant differences in biofilm production were observed among serotypes, though these associations were not consistent throughout the growth conditions. Moreover, lineage seemed to be a spurious indicator of biofilm formation in *L. monocytogenes* as both serogroups IIb and IVb belonging to lineage I, showed significant differences in biofilm formation under some conditions. Our results suggest that these criteria may not be relevant to reflect the actual intraspecific diversity of biofilm formation. Instead, other molecular markers such as MLST-defined genotypes could divulge stronger associations with phenotypes. Interestingly, recent epidemiological studies reported heterogeneous distribution of CCs among food and clinical *L. monocytogenes* isolates from which hyper- and hypovirulent genotypes were identified (Maury et al., 2016; Painset et al., 2019). Such approach on large collections of isolates did lead to conclusive findings. In particular, Maury et al. (2019) found that hypovirulent genotypes, CC121 and CC9, were more efficient in biofilm production under sub-lethal concentrations of benzalkonium chloride implying that certain genotypes may have fitness advantages under specific environmental conditions. Similarly, associations between genotypes and stress tolerance traits have been documented in *L. monocytogenes* (Hingston et al., 2017). In the current study, interestingly, three isolates of CC26 produced more biofilm than other CCs at 10°C. It is presumed that core genetic features of CC26 contributed to the exceptional behavior of these isolates under cold resulting in higher biofilm productivity. In contrast, high intra-genotype variations observed in some CCs suggest that minor genetic variants within a genotype may impact biofilm phenotype.

In addition, our data suggest that high ability to adhere to surface or to produce sessile biomass may not be a prerequisite to persistence or prevalence of *L. monocytogenes* in FPE. Other traits associated with stress factors in the food chain such as increased resistance to disinfectants or desiccation treatments need to be investigated to understand the factors stratifying the current population structure of *L. monocytogenes*. As a matter of fact, except for eight isolates of CC7 (persistent subgroup D) which revealed statistically indistinguishable phenotypes, other persistent clones (subgroup A, B, and C) expressed significantly varying biofilm phenotypes within each subgroup under some growth conditions. In the current study, persistence was defined when the same genotype was isolated at least three times from the same FPE or related food products over a timespan of more than 1 year. Evidently, there exists a limitation in subtyping methods such as PFGE or MLST for discriminating isolates belonging to the same subtype. Besides, it is difficult to discriminate the same clones regularly reintroduced into FPE from real persisters and likewise persistent clones could be mis-categorized as a sporadic isolate due to constraints in sampling schemes (NicAogáin and O’Byrne, 2016). In our study, isolates of the persistent subgroups were treated as individuals for analyses in order to maintain the similar numbers among the three groups, persistent, prevalent, and rare. Also, it was appropriate since phenotypic variations were observed within most of the subgroups. However, future studies involving thoroughly proven clones must consider the biases that can be introduced by analyzing the same isolates for multiple times. In such cases, the proper approach would be to select one isolate representative of each group of clones.

In the present study, basic experimental setups were designed to collect phenotypes suitable for pan-GWAS. Although temperature, nutrient availability, and osmolarity changes were integrated in this study, other environmental factors important during sessile growth were not addressed. More complex experimental designs, for instance, by integrating multi-species biofilms grown under hydrodynamic conditions (Giaouris et al., 2013; Puga et al., 2018) may approximate better to reality. However, collecting reproducible data from a large number of isolates would be challenging under such conditions. The pan-GWAS on total biomass produced under various growth conditions identified numerous genes from the accessory genome of 57 strains. Generally, the number of shared genes among different growth conditions was low due to high genomic variation. However, GO enrichment revealed that the distribution of modules of genes was comparable regardless of the growth conditions suggesting that overall patterns of genes involved in biofilm production were homogeneous.

Several studies investigated putative biofilm determinants using transposon mutagenesis (Chang et al., 2012; Yong et al., 2012; Alonso et al., 2014; Piercey et al., 2016). Among a wide spectrum of functions reported, “cell wall component” (teichoic acid metabolism and peptidoglycan synthesis), “membrane proteins” (lipoprotein assembly), “flagella and motility,” “cell signaling,” “energy generation and intermediary metabolism,” “biosynthesis,” and “gene regulations” were the most prevalent ones. Genes encoding virulence-related surface proteins such as Internalins and cell wall-anchored proteins were also found to be engaged in biofilm formation (Jordan et al., 2008; Franciosa et al., 2009; Popowska et al., 2017). Similar findings were observed among the genes identified by pan-GWAS in the current study. For example, functions including “cell surface proteins,” “soluble internalins,” and “cell wall” were overrepresented.

Interestingly, functional category “metabolism of phosphate” was enriched in the set of genes. Previously, phosphate-dependent biofilm production was suggested in *Pseudomonas fluorescens* (Navarro et al., 2011; Newell et al., 2011). Variations in the concentration of phosphate in the surrounding environment modulated expression of surface adhesin through a cyclic dimeric GMP (c-di-GMP) signaling pathway mediating the switch between planktonic and biofilm lifestyles. As an important second messenger, c-di-GMP plays a role in the complex regulation of broad bacterial behaviors including biofilm formation and exopolysaccharide synthesis in *L. monocytogenes* and in a wide variety of bacteria (Köseoğlu et al., 2015; Hengge et al., 2016; Valentini and Filloux, 2016). Phosphate is incorporated in nucleic acids and phospholipids which take up a substantial part of the cell. However, phosphate metabolism and the effect of phosphate on specific phenotypes has not been studied in depth.

Another highly enriched function was “transformation/competence.” Horizontal gene transfer and release of extracellular DNA is interconnected with quorum sensing during biofilm formation (Madsen et al., 2012; Ibáñez de Aldecoa et al., 2017). In *Streptococci*, biofilm maturation was dependent on allelic variants of *comC*, the gene encoding the competence-stimulating peptide and extracellular DNA played a crucial role in biofilm formation (Carrolo et al., 2010, 2014). Moreover, competence and transformation efficiencies were significantly induced during sessile growth accompanied by upregulation of competence genes (Marks et al., 2012). In *L. monocytogenes*, interaction of extracellular DNA with peptidoglycan was involved in adhesion (Harmsen et al., 2010). In this regard, the mechanism of interplay between cellular competence and extracellular DNA, an essential component of *L. monocytogenes* biofilm matrix, during its sessile life needs to be elucidated.

However, a limitation of the pan-GWAS approach was the lack of straightforward associations of presence/absence profile of genes with strong/weak biofilm formers because the occurrence of genes between two binary traits was obscure in general (average 29%). This may be explained by the difficulty in converting a linear trait into binary format which may have affected the results. Secondly, biofilm formation is a string of simultaneous processes engaging various steps as well as physiologically diverse cellular states. The number of genes identified by pan-GWAS may reflect this complexity. Transcriptomic studies conducted on sessile cells pointed out time-dependent differential gene expression patterns in *L. monocytogenes* and other bacteria (Stanley et al., 2003; Yamamoto et al., 2011; Tirumalai and Prakash, 2012). It supports the hypothesis that divergent modules of genes are involved at each step of biofilm formation. Thus, engagement of a gene in one step of biofilm formation might have been masked by its irrelevance during the other steps which might affect pan-GWAS results.

## Conclusion

This study is the first attempt to correlate biofilm traits with persistence status, frequency and distribution of genotypes as well as genetic composition in a well-characterized collection of food-related *L. monocytogenes* isolates. Under our laboratory experimental conditions, persistence or prevalence did not correspond to a higher biofilm formation capacity. The current distribution of genotypes in FPE and foods may be comparable to their heterogeneous distribution in natural habitats which, unfortunately, is difficult to investigate due to the lack of appropriate databases. Biofilm productivity exhibited profound inter-strain variations depending on growth conditions which resulted in inconsistent associations between biofilm phenotype and lineages or serotypes throughout the different conditions. Interestingly, a temperature-dependent association of genotype with biofilm production was observed asserting genotype as a better predictor of bacterial phenotypes. While salt addition enhanced biofilm production, nutrient deprivation impaired it. Importantly, a marked non-linear effect of both treatments was documented for the first time in *L. monocytogenes*. Supplementation of NaCl in nutrient deprived cells significantly increased biofilm maturity regardless of temperature. To better explain the observations encountered in the study, intraspecific variations in planktonic and sessile modes of growth under each condition shall be evaluated. Pan-GWAS was successfully applied on biofilm data and the module of genes were found to be comparable across different growth conditions in spite of the small number of shared genes. Cell surface proteins and transformation/competence-related functions were highly enriched among the total list of identified genes. Further investigations on genes of unknown function as well as a time-course omics approaches such as transcriptomics and proteomics will help decipher the complex mechanisms of biofilm formation.

## Data Availability Statement

The datasets generated for this study can be found in the https://www.ebi.ac.uk/ena/data/view/PRJEB15592 and http://www.ebi.ac.uk/ena/data/view/PRJEB32254.

## Author Contributions

B-HL and PP conceived the project and wrote the manuscript. B-HL designed the experiments, and analyzed and interpreted the data. SB-B, MH, and TB supervised the experiments concerning biofilm phenotypes conducted by SC and B-HL. LG performed the phenotype clustering and the pan-GWAS. NK performed annotation on EGDe. BF selected the strains. IS performed functional analysis. All authors reviewed and approved the final manuscript.

## Conflict of Interest

B-HL, SC, SB-B, and TB were employed by BioFilm Control SAS. B-HL and NK were employed by GenXPro GmbH. The remaining authors declare that the research was conducted in the absence of any commercial or financial relationships that could be construed as a potential conflict of interest.

## Footnotes

1 https://www.ebi.ac.uk/ena/data/view/PRJEB15592

2 http://www.ebi.ac.uk/ena/data/view/PRJEB32254

3 https://www.uniprot.org/

4 http://pantherdb.org/

5 http://genolist.pasteur.fr/ListiList/
