# Supplementary material for: Biofilm Formation of Listeria monocytogenes Strains Under Food Processing Environments and Pan-Genome-Wide Association Study
Source: Front Microbiol. 2019 Nov 21;10:2698. doi: 10.3389/fmicb.2019.02698 (PMC6882377; doi:10.3389/fmicb.2019.02698)
Supplement: Supplementary file 1 [file Data_Sheet_1.pdf]

Supplementary Figures

Biofilm formation of *Listeria monocytogenes* strains under food processing environments and pan-genome-wide association study

Bo-Hyung Lee[1,2,3]*, Sophie Cole[2], Stéphanie Badel-Berchoux[2], Laurent Guillier[4], Benjamin Felix[4], Nicolas Krezdorn[3], Michel Hébraud[5], Thierry Bernardi[2], Ibrahim Sultan[6], Pascal Piveteau[7]*

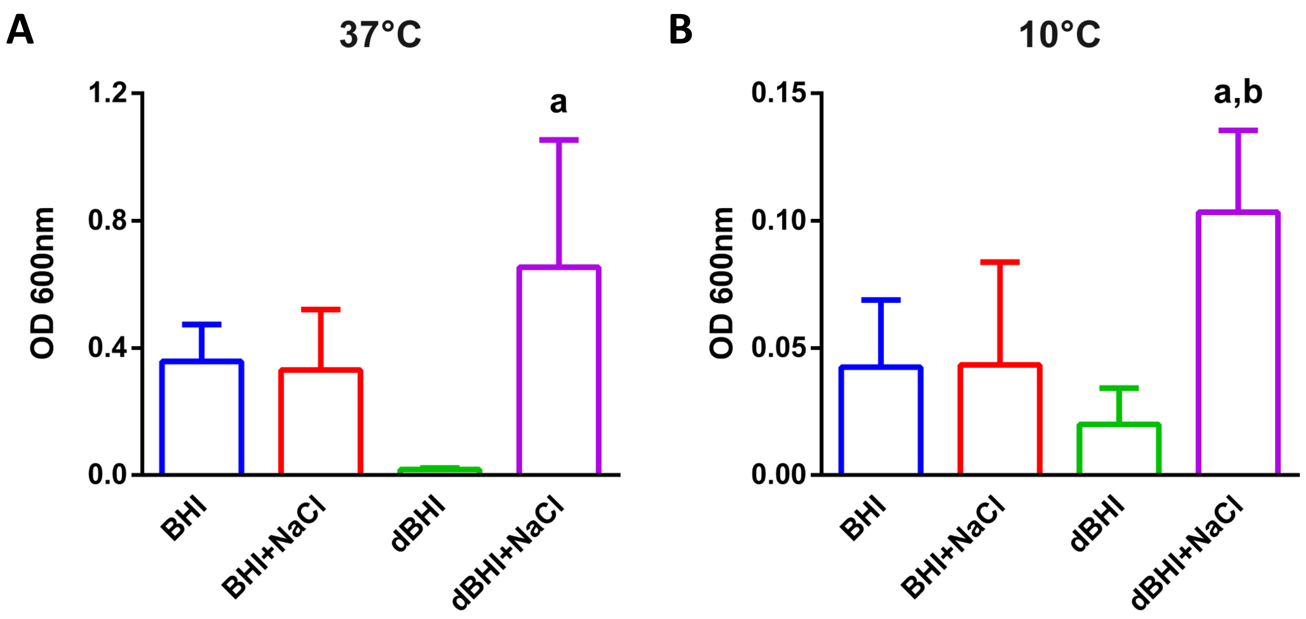

Supplementary Fig. 1. Total biomass of strain 13 (persistent subgroup D) quantified by MPA. Data are presented as mean + SD and statistical significance was assessed using One-way ANOVA and Dunnett's multiple comparison tests, a, $p < 0.01$ compared to dBHI; b, $p < 0.05$ compared to BHI.

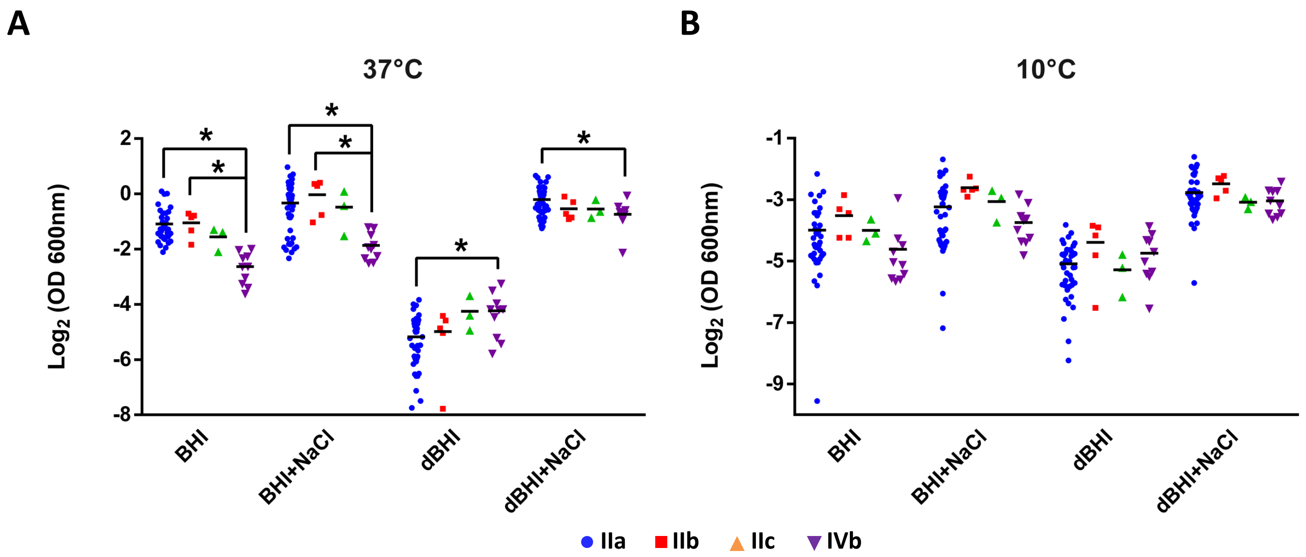

Supplementary Fig. 2. Comparative analysis of biofilm formation among serogroups. Total biomass obtained by MPA at (A) 37°C and (B) 10°C are compared between serogroups. Each dot represents an isolate and the horizontal bar the mean. Data were analyzed using One-way ANOVA and Turkey's multiple comparison tests, *, $p < 0.05$.

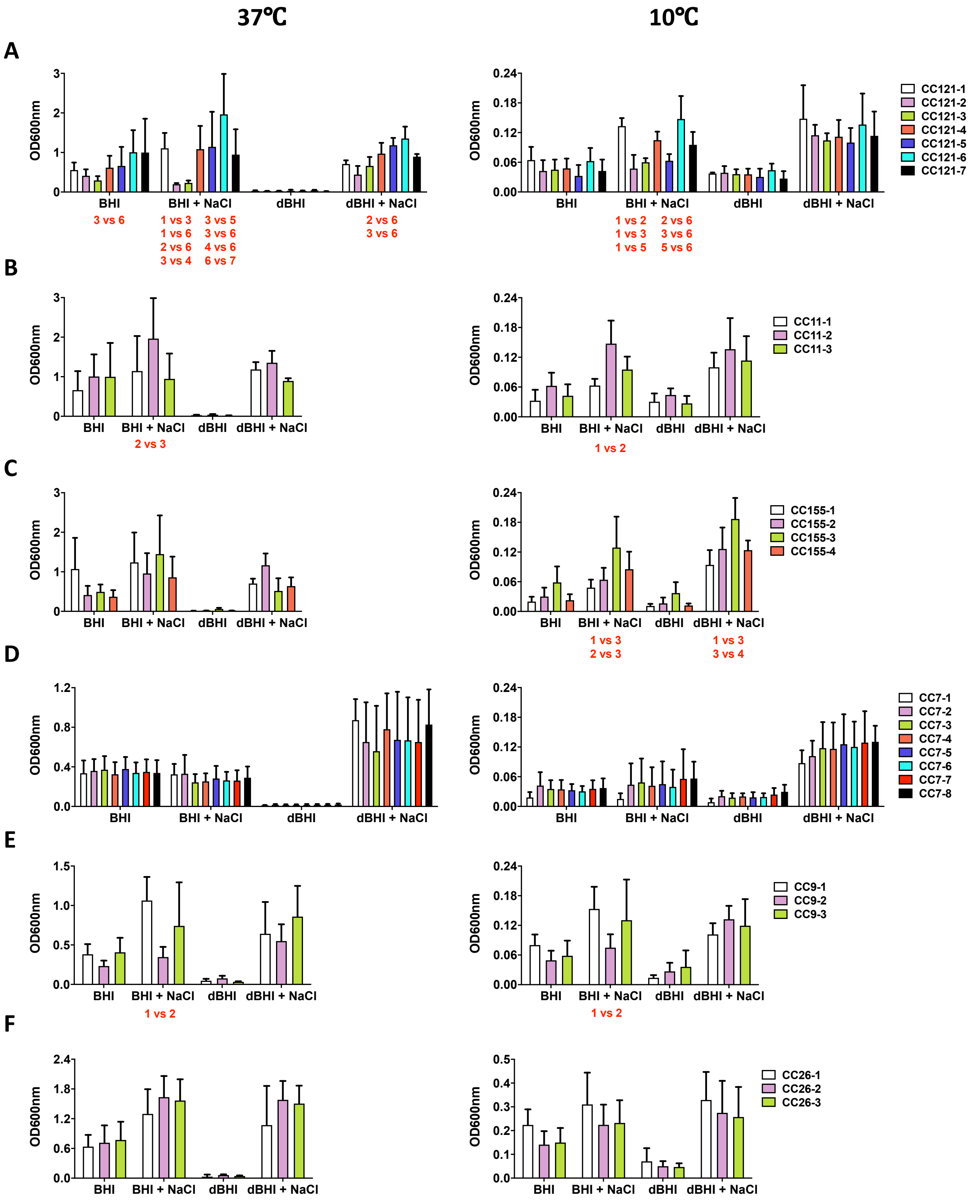

Supplementary Fig. 3. Intra-genotype comparison of biofilm formation. MPA results of isolates belonging to the same subtype are presented as mean + SD. Graphs on the left for 37°C and right for

10°C of (A) CC121, (B) CC11, (C) CC155, (D) CC7, (E) CC9, and (F) CC26. The number following the hyphen indicates the strains within each genotype in the legend. Data were analyzed using One-way ANOVA and Tukey's multiple comparisons test. Pairs of strains showing significant differences are noted under each growth condition, $p < 0.05$.
